# Effects of mindfulness-based stress reduction on cancer-related fatigue in patients with breast cancer: a meta-analysis of randomized controlled trials

**DOI:** 10.3389/fonc.2024.1425563

**Published:** 2024-10-03

**Authors:** XiaoQian Lan, HongMei Xie, Lan Fu, WenTao Peng

**Affiliations:** ^1^ West China School of Nursing, Sichuan University/Department of Anesthesiology, West China Hospital, Sichuan University, Chengdu, China; ^2^ West China School of Nursing, Sichuan University/Department of General Surgery, West China Hospital, Sichuan University/Breast Disease Center, West China Hospital, Sichuan University, Chengdu, China; ^3^ Department of Nursing, West China Second University Hospital, Sichuan University/West China School of Nursing, Sichuan University/Key Laboratory of Birth Defects and Related Diseases of Women and Children (Sichuan University), Ministry of Education, Chengdu, China

**Keywords:** mindfulness-based stress reduction, cancer-related fatigue, breast cancer, anticancer treatment, meta-analysis

## Abstract

**Introduction:**

Mindfulness-based stress reduction (MBSR) has been widely used for improving psychological symptoms and sleep quality in breast cancer patients and has a positive impact on posttraumatic growth and immunology. Moreover, MBSR is increasingly being used in cancer-related fatigue (CRF) intervention studies for breast cancer patients, but conflicting results also exist.

**Objective:**

This study aimed to evaluate the effect of MBSR on CRF in patients with breast cancer.

**Methods:**

A comprehensive computer search of the Pubmed, Cochrane Library, Embase, Web of Science, China Biomedical Document Service System, China Knowledge Infrastructure Engineering, Wanfang Data Knowledge Service Platform, and VIP databases was performed. Randomized controlled trials (RCTs) published before April 10, 2023, were identified. The primary outcome was cancer-related fatigue associated with breast cancer. Two researchers independently screened the studies, extracted the data, and evaluated the methodological quality of the studies according to the inclusion and exclusion criteria. The Meta-analysis of the outcome indicators was performed using STATA 16.0 software.

**Results:**

A total of 13 studies were included, including 1992 patients (997 patients in the MBSR group and 1015 patients in the control group). Compared with conventional care, MBSR significantly alleviated the symptoms of CRF in breast cancer patients (SMD=-0.32, 95% CI [-0.42, -0.22], z=6.54, p<.01). Under the supervision of experts, the 8-week MBSR had a great influence on CRF, especially in the Asian population.

**Conclusions:**

MBSR is effective in the treatment of CRF induced by breast cancer, and no obvious adverse effects occur; thus, MBSR can be recommended as a beneficial adjuvant therapy for treating CRF in breast cancer patients.

**Systematic review registration:**

https://www.crd.york.ac.uk/prospero/, identifier CRD42021245365.

## 1 Introduction

Breast cancer is one of the most common malignant tumors that affects the cells of the breast. In 2020, it was estimated that 2.3 million women will be diagnosed with breast cancer, accounting for 11.7% of all new cancer diagnoses, which has surpassed the number of new lung cancers and become the most commonly diagnosed cancer among females worldwide (1). The incidence of breast cancer has been increasing in recent years; however, its mortality rate accounts for only 6.9% of cancer deaths (1). Moreover, approximately 70-80% of patients who suffer from early nonmetastatic disease will eventually be cured (2). The 5-year survival rate of patients with breast cancer in developed countries has exceeded 85%, which is related to advancements in medical technology and the early detection, diagnosis, and treatment of breast cancer (3). Despite great progress in improving the survival rates of patients with breast cancer in recent years, the burden of various symptoms (such as nausea, vomiting, and fatigue), particularly those caused by surgery, radiotherapy, and chemotherapy, affects the quality of life of patients. Therefore, formulating management strategies for related symptoms is an important part of oncology research. At present, the burden of symptoms such as nausea and vomiting is better managed, but cancer-related fatigue poses great challenges for the treatment of this severe problem (2, 4).

Cancer-related fatigue (CRF) has been documented as one of the most common and distressing symptoms of cancer patients and survivors, particularly in breast cancer survivors (5). Studies on CRF in breast cancer patients have shown that 56% of breast cancer patients who receive endocrine therapy have CRF symptoms (6, 7). Compared with other treatments, the risk of fatigue increases after receiving a combination of surgery, chemotherapy, and radiotherapy (RR 1.18, 95% CI 1.05-1.33), and the prevalence of severe fatigue is between 7% and 52% and may continue for months or years after treatment is completed. There is increasing evidence demonstrating that a quarter of breast cancer survivors will experience fatigue even up to 10 years postdiagnosis (8). This kind of fatigue can be differentiated from simple tiredness, which cannot be relieved by sleeping or resting and is a lasting subjective physical, emotional or cognitive fatigue or fatigue feeling that may interfere with normal function, leading to a decrease in the clinical treatment effect and treatment compliance of patients, affecting the prognosis of patients, and decreasing the overall quality of life (9). In addition, cancer survivors with CRF symptoms may be less involved in employment and face high levels of economic pressure (10, 11). Moreover, cancer patients use medical resources more frequently, and CRF may also reduce their survival rate (12, 13). Therefore, strategies for improving CFR symptoms in breast cancer patients are highly warranted. However, the etiology and pathogenesis of CRF have not been fully characterized to date. The reasons may be related to changes in muscle metabolism caused by reduced physical activity, cytokine disorders, hypothalamic-pituitary-adrenal axis destruction and circadian rhythm disorders, genetic risk factors, and psychological and biological behavior-related risk factors (14–16). Hence, it is an enormous challenge to address effective CRF prevention and treatment in this context. The National Comprehensive Cancer Network (NCCN) guidelines suggest that the treatable factors that may contribute to fatigue should be treated first (17). The National Comprehensive Cancer Network (NCCN) and the European Society for Medical Oncology (ESMO) recommend drug or nondrug treatment as common clinical treatments. The EMSO summarizes the latest intervention measures for CRF treatment (18). Drug treatments include antidepressants, hormones, and psychostimulants, while nondrug treatments include moderate-intensity physical exercise, resistance exercise, psychotherapy, physical and mental intervention (MBSR), and acupuncture. Currently, there are no specific drugs available for the treatment of CRF. In addition, physical exercises and resistance exercises are not suitable for patients during the treatment period, and acupuncture treatment remains controversial (19). Mindfulness-based stress reduction (MBSR), a nonpharmaceutical intervention, is considered to be beneficial for reducing CRF and minimizing its impact on patient function and is worthy of further study (20, 21). The MBSR is a group-based 8-week program proposed by Professor Jon Kabat-Zinn of the University of Massachusetts Medical Center that includes body scanning, mindfulness meditation, mindfulness exercise and psychological education (22). The purpose of MBSR is to guide patients to cultivate mindfulness by using their physical and mental strength, making self-adjustments, enhancing patients’ ability to cope with diseases, and improving their quality of life and prognosis.

In recent years, MBSR has been widely used in breast cancer patients to reduce psychological symptoms and improve sleep quality and has a positive impact on posttraumatic growth and immunology (23–25). There is a relationship between MBSR and changes in gray matter concentration in brain regions that regulate emotion, self-referential processing, and learning and memory processes (26). The effects of MBSR on the brain and the immune system are important, particularly in understanding how the brain processes emotions under stress (26). Several studies have indicated a beneficial relationship between stress reduction and quality of life (QoL), along with simultaneous improvements in the immune system following MBSR (21, 27, 28). MBSR significantly improves mood disturbances, anxiety, anger, vigor, fatigue, confusion, endocrine and breast-related quality of life, physical well-being, and general well-being by reducing rumination and reactions to emotional and physical triggers in breast cancer survivors (29, 30). MBSR is increasingly being used in CRF intervention studies for breast cancer patients, but conflicting results also exist (31). In addition, the sample size in the previous studies was relatively small, and it may be difficult to obtain a relatively accurate conclusion based on a single study (32–36). Although some systematic reviews and meta-analyses have also proven that MBSR can improve the symptoms of CRF in cancer patients (37, 38), there is no meta-analysis of the efficacy of MBSR on CRF in breast cancer patients. Therefore, this study aimed to explore the effect of MBSR on CRF in breast cancer patients through a meta-analysis and to provide evidence for its use in clinical practice.

## 2 Materials and methods

### 2.1 Search strategy

Studies on the effect of MBSR on the CRF of breast cancer patients in the PubMed, National Library of Medicine, Web of Science, Embase, Cochrane Library, China National Knowledge Infrastructure, Chinese Biomedical Study Database, Wanfang Data Knowledge Service platform and VIP databases were included in this research. The retrieval time was set to the time of database-building on April 10, 2023. The search was carried out by the combination of the following keywords and Medical Subject Headings (MeSH) terms: (Breast Neoplasm OR Neoplasm, Breast OR Breast Tumors OR Breast Tumor OR Tumor, Breast OR Tumors, Breast OR Neoplasms, Breast; Mesh term: Breast Neoplasms) and (mindfulness-based stress reduction or Mindfulness-Based Stress Reduction or Mindfulness or mindfulness-based stress reduction or MBSR or meditation or relaxation therapy or mind-body therapies; MeSH term: Mindfulness) and (Lassitude or fatigue cancer related or cancer-related fatigue or Asthenia; MeSH term: Fatigue). The search strategy was slightly adjusted according to the characteristics of the different databases without restriction on language. The references included in the studies were manually searched by the “snowball” method to improve the study recall rate, and the published references of related systematic reviews and meta-analyses were traced back to obtain unsearched related studies. The search strategy for PubMed is described in detail in **Supplementary File 1**.

### 2.2 Inclusion and exclusion criteria

Enrolled studies were required to meet the following criteria: (1) the study design was a randomized controlled trial (RCT); (2) the study subjects were histopathologically diagnosed with breast cancer, aged ≥18 years, and patients who had completed or were receiving adjuvant therapy; (3) the intervention and control measures of the studies were MBSR intervention and usual care, respectively; and (4) the outcome indicators of the studies were complete CRF fatigue scores (mean ± standard deviation).

Reports were excluded if they met one of the following criteria: (1) were a duplicate published study; (2) had no access to the full text; (3) had missing outcome indicators; (4) were case reports, conferences, or review studies; (5) had incomplete data or inability to extract valid data from the study; or (6) had an unreasonable research design or poor-quality study.

### 2.3 Study screening and data extraction

**Figure 1:**
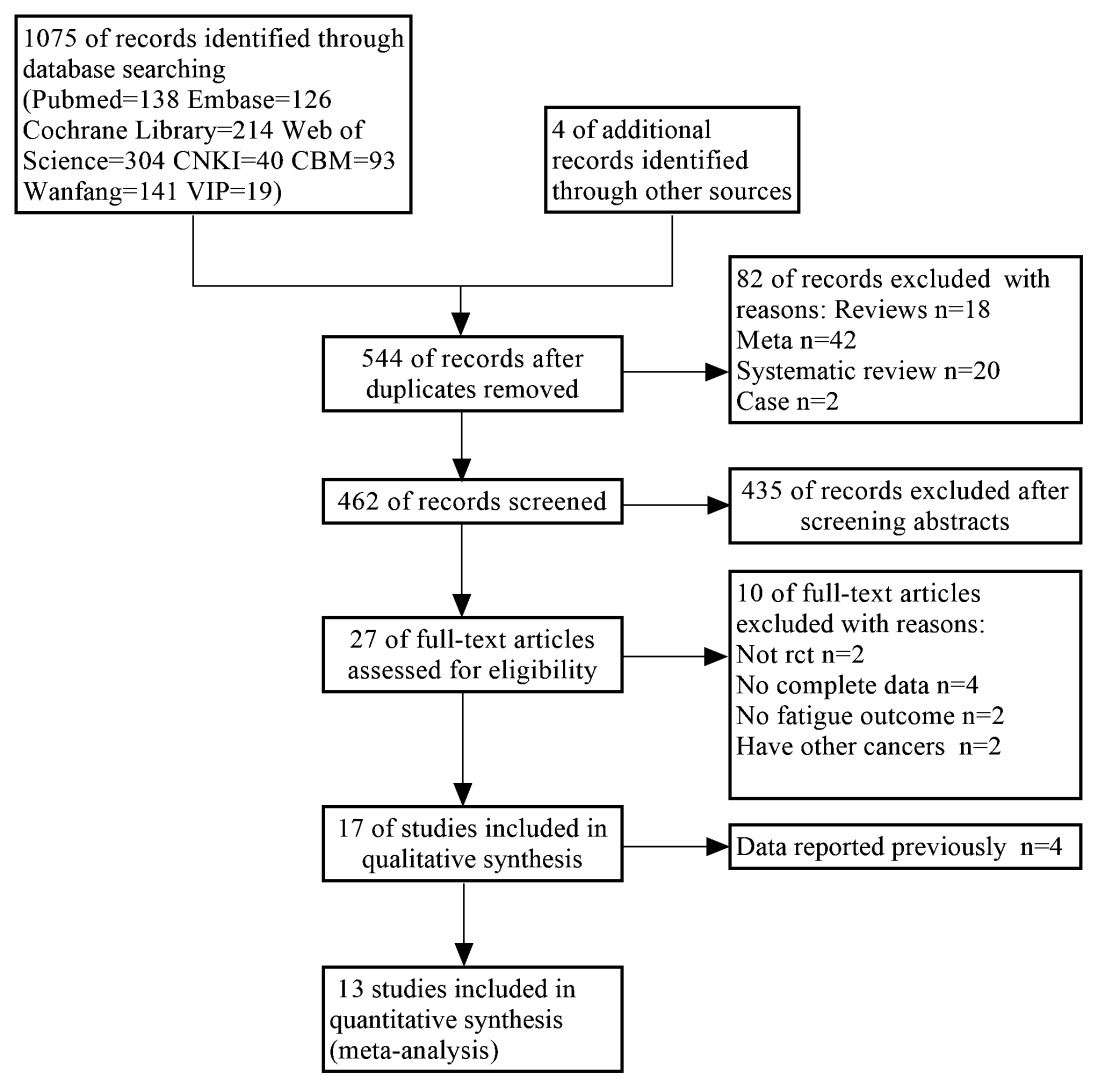
Results of the study search and screening procedures.

Based on the inclusion and exclusion criteria, document screening, quality assessment of methodology, and data extraction were conducted by 2 researchers separately. Any areas of disagreement were discussed until a consensus was reached or adjudicated by a third researcher. As shown in **Figure 1**, a total of 1079 references were retrieved. After the elimination of duplicate studies, reviews, systematic reviews, meta-analyses and case reports, 462 unique references were identified. After the titles and abstracts were read, 27 studies were obtained for full-text screening. After the full texts were read, 13 studies were ultimately included. A unified data extraction table was used for data extraction, including first author, publication year, country/region, research design, sample size, general characteristics of subjects (age and sex, disease stage, treatment stage), characteristics of intervention measures and control measures (intervention form, time and frequency), and outcome indicators.

### 2.4 Risk assessment of bias in the included studies

Two independent reviewers evaluated the included studies and cross-checked the results according to the bias risk assessment tool recommended by the Cochrane Handbook (5.1.0). If there was any difference of opinion, the third researcher was asked to arbitrate and reach a consensus. The evaluation included the following 7 items: (1) random sequence generation; (2) allocation concealment; (3) blinding of participants and personnel; (4) blinding of outcome assessment; (5) incomplete outcome data; (6) selective reporting; and (7) other bias.

### 2.5 Statistical analysis

The meta-analysis was performed using STATA software 16.0. First, the heterogeneity of the data was analyzed, and a value of p > 0.1 for the Q test or I2 < 50% indicated low heterogeneity among the studies. The fixed-effect model was used for the merging of homogeneous data. A value of p < 0.1 for the Q test or I2 ≥ 50% indicated significant heterogeneity among the studies. The source of the heterogeneity was further analyzed. If there was no clinical heterogeneity, the random effects model was used to perform the meta-analysis. In this study, only quantitative data were included. If the same scale was used, the mean difference (MD) was selected as the effect index. Otherwise, the standardized mean difference (SMD) was used. All effect quantities are represented as 95% confidence intervals (95% CIs). Egger’s test was used to detect potential publication bias, and p > 0.05 was considered to indicate no publication bias.

## 3 Results

### 3.1 Basic characteristics of the included studies

A total of 13 randomized controlled trials were included, with a total of 1992 patients, including 977 patients in the experimental group and 1015 patients in the control group (28, 30, 32–36, 39–44). The sample size ranged from 24 to 299, with a median ‘n’ of 95, an average age of 35.7 to 58.1 years and a median age of 52.6 years. The experimental group in the 16 studies adopted MBSR, while the control group adopted usual care. The intervention time of 4 studies was 6 weeks (33.34.41.43), and that of 9 studies was 8 weeks (28, 32, 35, 36, 39, 40, 42–44). The study samples were from the United States, Canada, the United Kingdom, China, Iran, and South Korea, and the subjects of the study were mainly stage 0-III nonmetastatic breast cancer patients. 5 studies included breast cancer patients who underwent chemotherapy, radiotherapy or surgery (32, 35, 39, 40, 42), and 8 studies included breast cancer patients who had finished treatment for a while (15, 28, 30, 33, 34, 41, 43, 44). 1 study adopted the PFS-R (39), 3 studies adopted the CFS (35, 36, 42), 2 studies adopted the POMS (27, 43), 2 studies adopted the MDASI (33, 34), 2 studies adopted the FSI (30, 41), 1 study adopted the subscale fatigue of the QLQ-C30 (43), 1 study adopted the FSS (32), and 1 study adopted the MFSI-SF (40). The duration of the intervention ranged from 6-8 weeks. The basic characteristics of the included studies are shown in **Table 1**.

**Table 1:** Basic characteristics of the included studies.

Author, Year, Country	Sample		Age		Cancer stage	Current treatment stage	Intervention		Assessment time	Fatigue scale	Adverse events
	Treatment	Control	Treatment	Control			Treatment	Control			
Hoffman et al. (2012) UK (28)	103	111	49.0 ± 9.2	50.1 ± 9.1	0- III	off-treatment	MBSR classes,8 weekly classes of 2 h in length except the first and last classes were 2.25 h in length, plus one 6-h day of mindfulness in week 6	UC	Baseline, 8 weeks, 18 weeks	POMS	none
Lengacher et al. (2012) USA (34)	41	43	58.0 ± 9.4		0- III	off-treatment	MBSR classes,6 weeks, 2 h/(time/week)	UC	Baseline, 6 weeks	MDASI	NA
Carlson et al. (2013) Canada (44)	113	54	54.6 ± 9.7	56.2 ± 10.8	I- III	off-treatment	MBSR classes,8 weeks, 2 h/(time/week)	UC	Baseline, 8 weeks	POMS	NA
Reich et al. (2014) USA (33)	17	24	58.0 ± 10.3	58.2 ± 9.5	0- III	off-treatment	MBSR classes,6 weeks, 2 h/(time/week)	UC	Baseline, 6 weeks	MDASI	NA
Rahmani et al (2015). Iran (32)	12	12	43.2 ± 3.0	44.0 ± 3.2	I- III	Surgery, chemotherapy, radiotherapy and other treatment	MBSR classes,8 weeks, 2 h/(time/week)	UC	Baseline, 8 weeks, 3 months	FSS	NA
Jang et al. (2016) Korea (43)	12	12	51.7 ± 5.3	51.4 ± 6.3	0- III	off-treatment	MBSR classes,8 weeks, 2 h/(time/week)	UC	Baseline, 8 weeks	QLQ-C30	NA
Lengacher et al (2016). USA (30)	152	147	56.5 ± 10.2	57.6 ± 9.2	0- III	off-treatment	MBSR classes,6 weeks, 2 h/(time/week)	UC	Baseline, 8 weeks, 12 weeks	FSI	NA
CAO et al. (2016) China (42)	100	100	36.1 ± 9.6	35.4 ± 9.2	I- III	chemotherapy	MBSR classes,8 weeks, 45-60 minutes each time	UC	Baseline, 8 weeks	CFS	NA
Reich et al. (2017) USA (41)	152	145	56.6		0- III	off-treatment	MBSR classes,6 weeks, 2 h/(time/week)	UC	Baseline, 8 weeks, 12 weeks	FSI	NA
Janusek et al. (2019) USA (40)	63	61	55.0 ± 10.1	55.2 ± 10.1	0- III	Surgery, chemotherapy, radiotherapy and other treatment	MBSR classes,8 weeks, 2 h/(time/week)	UC	Baseline, 8 weeks, 3 months, 6 months	MFSI-SF	NA
SHEN et al. (2020) China (36)	37	35	36.2 ± 4.6	36.5 ± 5.0	NA	off-treatment	MBSR classes,8 weeks, 30-45 minutes each time, at least 6 days a week	UC	Baseline, 8 weeks, 3 months	CFS	NA
ZOU et al. (2020) China (35)	23	23	46.3 ± 7.5	46.1 ± 7.1	NA	chemotherapy	MBSR classes,8 weeks, 30 minutes each time, at least 5 days a week	UC	Baseline, 8 weeks	CFS	NA
FAN et al. (2021) China (39)	90	90	45.2 ± 5.4	46.3 ± 5.6	0- III	chemotherapy	MBSR classes,8 weeks, 2-3 h/time, once in the morning and once in the evening.	UC	Baseline, 8 weeks	PFS-R	NA

UC, usual care; MBSR, Mindfulness-Based Stress Reduction; POMS, Profile of Mood States; MDASI, M.D. Anderson Symptom Inventory; FSS, Fatigue Severity Scale; EORTC-QLQ-C30, European Organization for Research and Treatment Quality of Life Questionnaires; FSI, Fatigue Symptom Inventory; CFS, Cancer Fatigue Scale; MFSI-SF, Multidimensional Fatigue Symptom-Short Form; PFS-R, Revised Piper Fatigue Scale; NA, not available.

### 3.2 Methodological quality of the included studies

**Figure 2:**
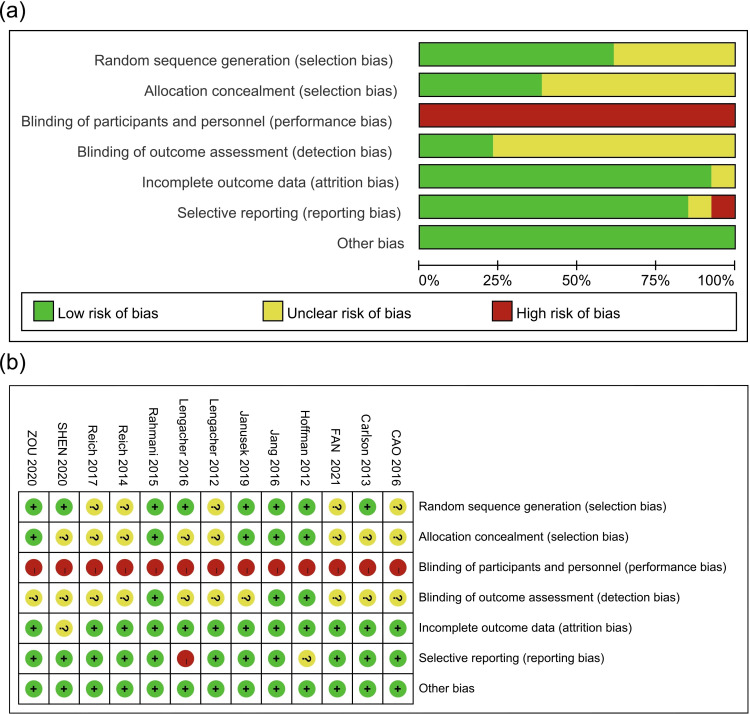
**(A)** Bias risk diagram; **(B)** Summary of risk of bias.

The Cochrane risk assessment tool was used to assess the quality of the included studies, and a bias risk diagram and bias risk summary chart were constructed, as shown in **Figures 2A, B**. All of the included studies were randomized clinical trials (RCTs), among which 8 studies adopted the correct randomization method. Five studies conducted allocation concealment with opaque envelopes. None of the 13 studies used blinding methods for the subjects or interveners, and only 3 studies used blinding methods for the evaluators of outcome indicators. Due to the difference between mindfulness decompression and routine nursing, it was impossible to blind the subjects and interveners, which may cause selection bias and measurement bias. One study had incomplete outcomes and did not perform an intention-to-treat analysis. One study had a selective result report, which was “high risk”, and another study might have a selective result report, which was “unclear”. All included studies had no other sources of bias.

### 3.3 Meta-analysis results

#### 3.3.1 Early effects of MBSR on CRF in breast cancer patients

**Figure 3:**
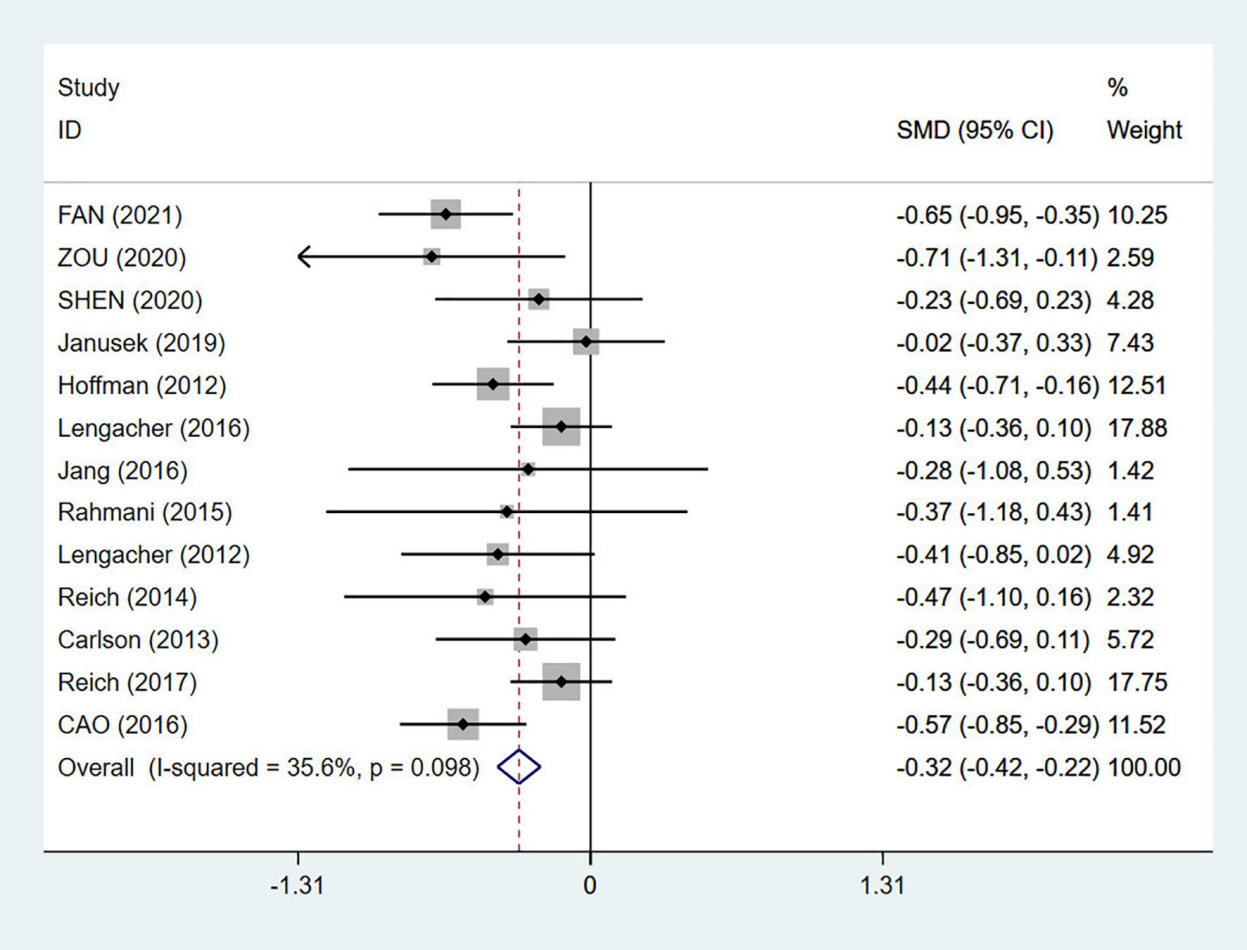
Forest plot of the early effects of mindfulness-based stress reduction (MBSR) on fatigue.

A total of 13 RCTs were included, with a total of 1992 patients (28, 30, 32–36, 39–44). After a heterogeneity test (I2 = 35.6%, p = 0.098), a fixed effects model was used for the meta-analysis. The results of the meta-analysis showed an SMD of -0.32 (95% CI: -0.42, -0.22), and p < 0.01. Compared with conventional nursing, MBSR had a statistically significant effect on early fatigue in breast cancer patients. After MBSR intervention, the fatigue symptoms of breast cancer patients improved immediately, as shown in **Figure 3**.

#### 3.3.2 Midterm effect of MBSR on CRF in breast cancer patients

**Figure 4:**
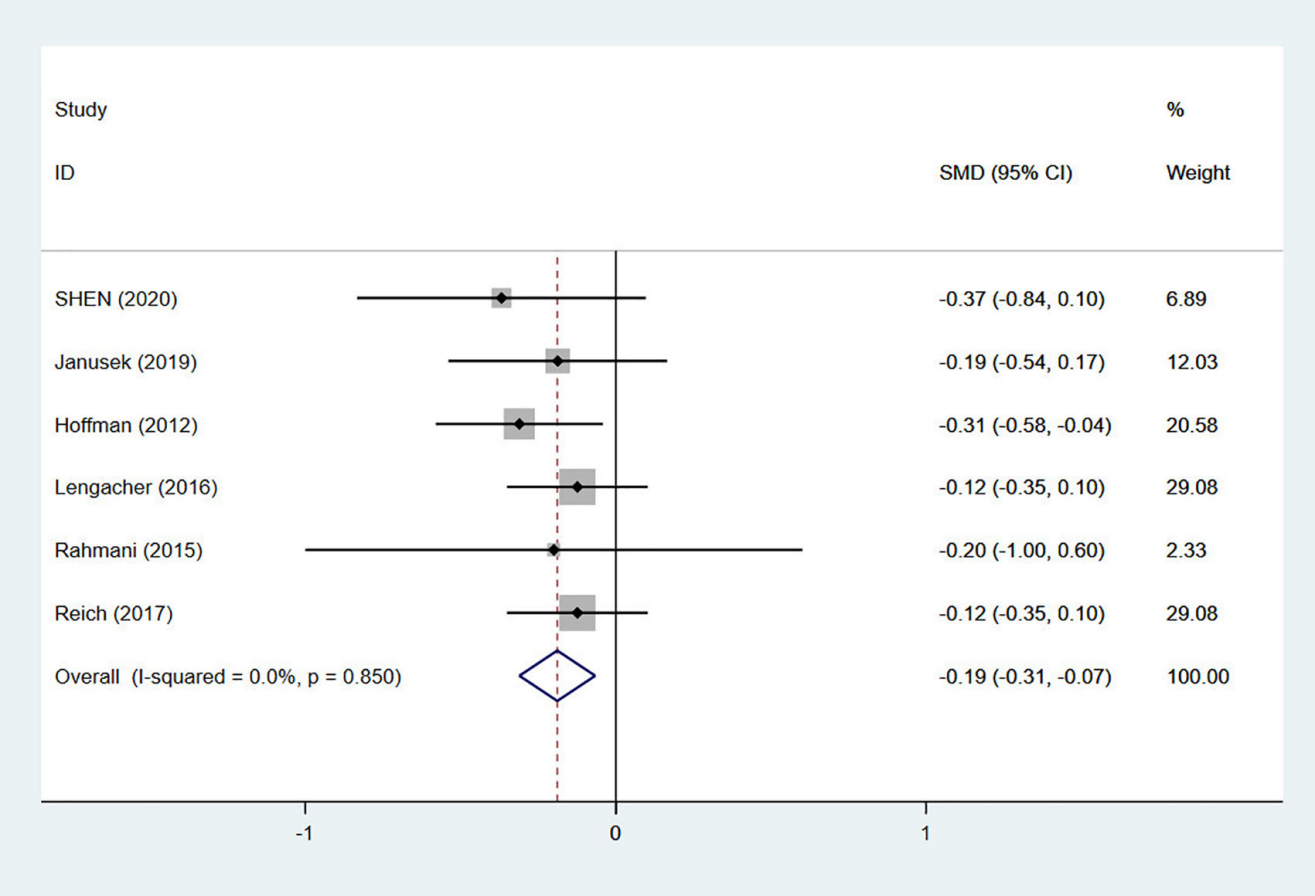
Forest plot of the medium-term effect of mindfulness-based stress reduction (MBSR) on fatigue.

A total of 6 studies performed follow-up evaluations 3 months to 6 months after MBSR intervention, with a total of 1030 patients (28, 30, 30, 32, 36, 40, 41). The pooled test showed low heterogeneity (I2 = 0%, p = 0.850), and a fixed effects model was used for the meta-analysis. The results of the meta-analysis showed an SMD of -0.19 (95% CI: -0.31, -0.07), and p < 0.01. Compared with that of conventional nursing, the midterm effect of MBSR was statistically significant, which indicated that the effect of MBSR on the fatigue of breast cancer patients could persist for 6 months after the intervention, as shown in **Figure 4**.

#### 3.3.3 Subgroup analysis of the effect of MBSR on the early follow-up of CRF

Results of sample size subgroup analysis: The total sample size for the 4 included studies was less than 50 (32, 33, 35, 43), and the results of the meta-analysis showed an SMD of -0.50 (95% CI: -0.84, -0.15) and p < 0.05. The total sample size for the 2 included studies ranged from 50 to 100 (34, 36), and the results of the meta-analysis showed an SMD of -0.33 (95% CI: -0.65, -0.01) and p < 0.05. The total sample size for the 7 included studies was more than 100 (28, 30, 39–42, 44), and the results of the meta-analysis showed an SMD of -0.30 (95% CI: -0.41, -0.20) and p < 0.05. Subgroup analysis of different sample sizes showed that CRF scores in the intervention group were lower than those in the control group.

Results of intervention methods subgroup analysis: The intervention method of 5 included studies was expert guidance (33, 35, 41, 42, 44), and the results of the meta-analysis showed an SMD of -0.34 (95% CI: -0.49, -0.19) and p < 0.05. The intervention method used in the 9 included studies was expert guidance combined with CD (24, 28, 30, 32, 36, 39, 40, 43), and the results of the meta-analysis showed an SMD of -0.31 (95% CI: -0.43, -0.18) and p < 0.05. Subgroup analysis of different intervention methods revealed that MBSR had a significant impact on fatigue, among which expert guidance of the MBSR intervention method had a greater impact on fatigue.

Results of the intervention time subgroup analysis: The intervention time of the 4 included studies was 6 weeks (30, 33, 34, 41), and the results of the meta-analysis showed an SMD of -0.18 (95% CI: -0.33, -0.04) (p < 0.05). The intervention time of the 9 included studies was 8 weeks (28, 32, 35, 36, 39, 40, 42–44), and the results of the meta-analysis showed an SMD of -0.42 (95% CI: -0.55, -0.30) and p < 0.05. Subgroup analysis of different intervention durations showed that MBSR had a statistically significant impact on fatigue, and MBSR for 8 weeks had a greater impact on fatigue.

Results of region subgroup analysis: Six studies were from Asia, including China, Iran, and South Korea (32, 35, 36, 39, 42, 43). The results of the meta-analysis showed an SMD of -0.54 (95% CI: -0.71, -0.37), and p < 0.05. One study was from the UK (28). The results of the meta-analysis showed an SMD of -0.44 (95% CI: -0.71, -0.16), and p < 0.05. In addition, six studies were from North America (30, 33, 34, 40, 41, 44). The results of the meta-analysis showed an SMD of -0.17 (95% CI: -0.30, -0.04) and p < 0.05. Subgroup analysis of different regions revealed that MBSR had a statistically significant impact on fatigue, and MBSR had a greater impact on fatigue in the Asian population.

Results of assessment tool subgroup analysis: Three studies adopted the CFS (35, 36, 42), and 2 studies adopted the POMS (28, 44). Subgroup analysis revealed that the CRF scores of the intervention group were lower than those of the control group [CFS scale: SMD = -0.51, (95% CI: -0.73, -0.29), p < 0.01; POMS scale: SMD = -0.39, (95% CI: -0.62, -0.17), p < 0.05]. The heterogeneity decreased significantly after subgroup analyses. Subgroup analysis of the effect of mindfulness-based stress reduction (MBSR) on early-stage CRF are shown in **Table 2**.

**Table 2:** Subgroup analysis of the effect of mindfulness-based stress reduction (MBSR) on early-stage CRF.

Subgroups	*K*	Sample size		Heterogeneity test		Fixed effect model					Random effect model			
		Treatment	Control	*P*	*I* ^2^	SMD	*95%CI*			*P*	SMD	*95%CI*		*P*
							L	U				L	U	
Sample size(n)														
n<50	4	64	71	0.833	0	-0.50	-0.84	-0.15		<0.05	-0.50	-0.84	-0.15	<0.05
50≤n ≤ 100	2	116	114	0.425	0	-0.33	-0.65	-0.01		<0.05	-0.33	-0.65	-0.01	<0.05
n>100	7	729	691	0.012	63.2	-0.30	-0.41	-0.20		<0.05	-0.32	-0.49	-0.14	<0.05
Intervention methods														
Expert guidance	5	361	329	0.112	46.6	-0.34	-0.49	-0.19		<0.05	-0.38	-0.61	-0.15	<0.05
Expert guidance and compact disc	8	548	436	0.106	39.3	-0.31	-0.43	-0.18		<0.05	-0.32	-0.48	-0.15	<0.05
Program length														
6 weeks	4	362	359	0.511	0	-0.18	-0.33	-0.04		<0.05	-0.18	-0.33	-0.04	<0.05
8 weeks	9	547	517	0.255	20.4	-0.42	-0.55	-0.30		<0.05	-0.41	-0.57	-0.26	<0.05
Region														
Asia	6	312	308	0.759	0	-0.54	-0.71	-0.37		<0.05	-0.54	-0.71	-0.37	<0.05
Europe	1	103	111	NA	NA	-0.44	-0.71	-0.16		<0.05	-0.44	-0.71	-0.16	<0.05
North America	6	494	457	0.642	0	-0.17	-0.30	-0.04		<0.05	-0.17	-0.30	-0.04	<0.05
Assessment tool														
PFS-R	1	90	90	NA	NA	-0.65	-0.95	-0.35		<0.05	-0.65	-0.95	-0.35	<0.05
CFS	3	160	158	0.368	0	-0.51	-0.73	-0.29		<0.05	-0.51	-0.73	-0.29	<0.05
MFSI-SF	1	63	61	NA	NA	-0.02	-0.37		0.33	0.911	-0.02	-0.37	0.33	0.911
POMS	2	172	148	0.556	0	-0.39	-0.62		-0.17	<0.05	-0.39	-0.62	-0.17	<0.05
FSI	2	304	292	1.0	0	-0.13	-0.29		0.03	0.111	-0.13	-0.29	0.03	0.111
QLQ-C30	1	12	12	NA	NA	-0.28	-1.08		0.53	0.497	-0.28	-1.08	0.53	0.497
FSS	1	12	12	0	0	-0.37	-1.18		0.43	0.364	-0.37	-1.18	0.43	0.364
MDASI	2	58	67	0.883	0	-0.43	-0.79		-0.08	<0.05	-0.43	-0.79	-0.08	<0.05

K, number of studies; SMD, standardized mean difference effect size; L, lower; U, upper; NA, not available.

**Figure 5:**
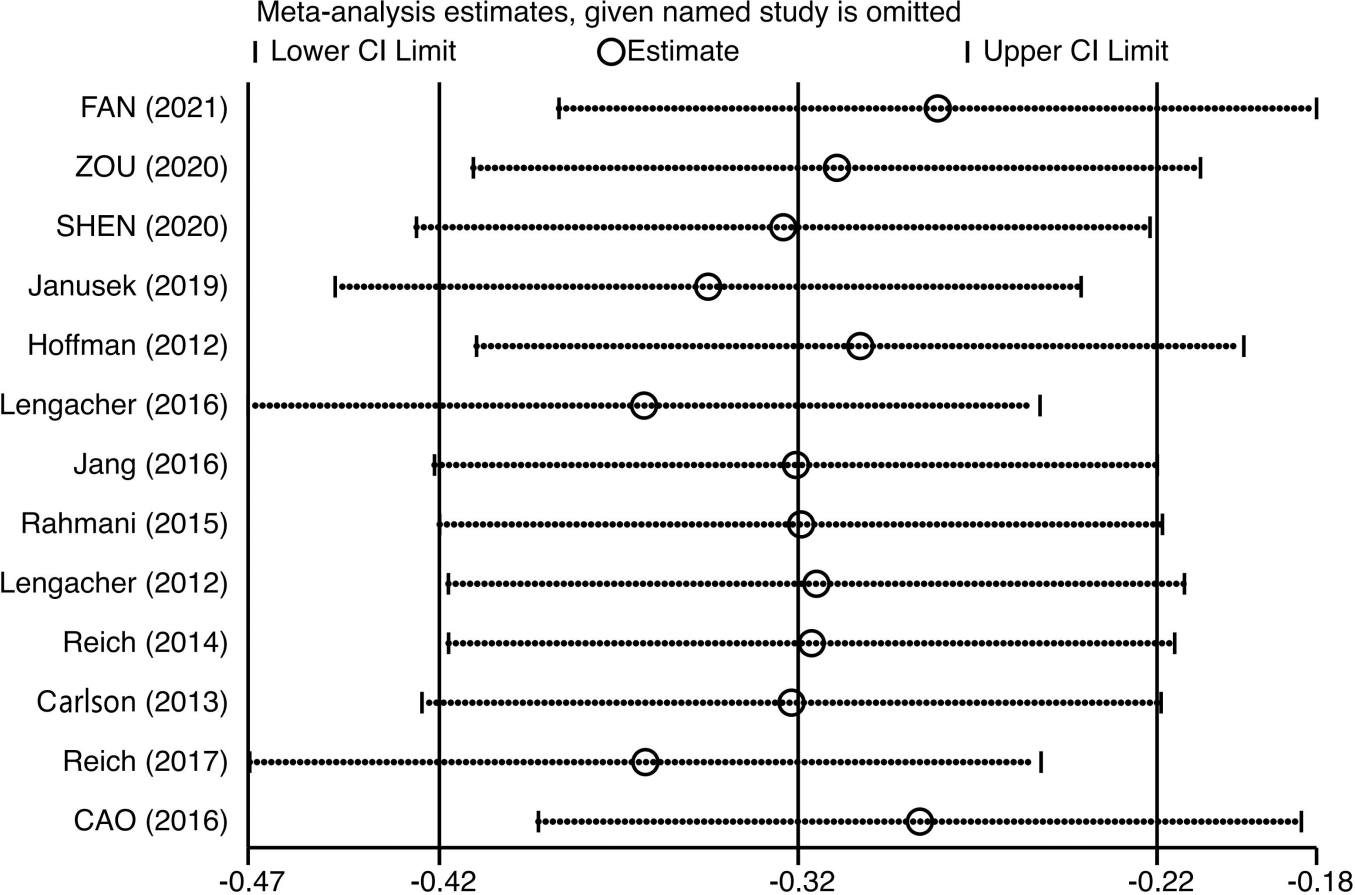
Sensitivity analysis of the effect of mindfulness-based stress reduction (MBSR) on fatigue in breast cancer patients.

**Figure 6:**
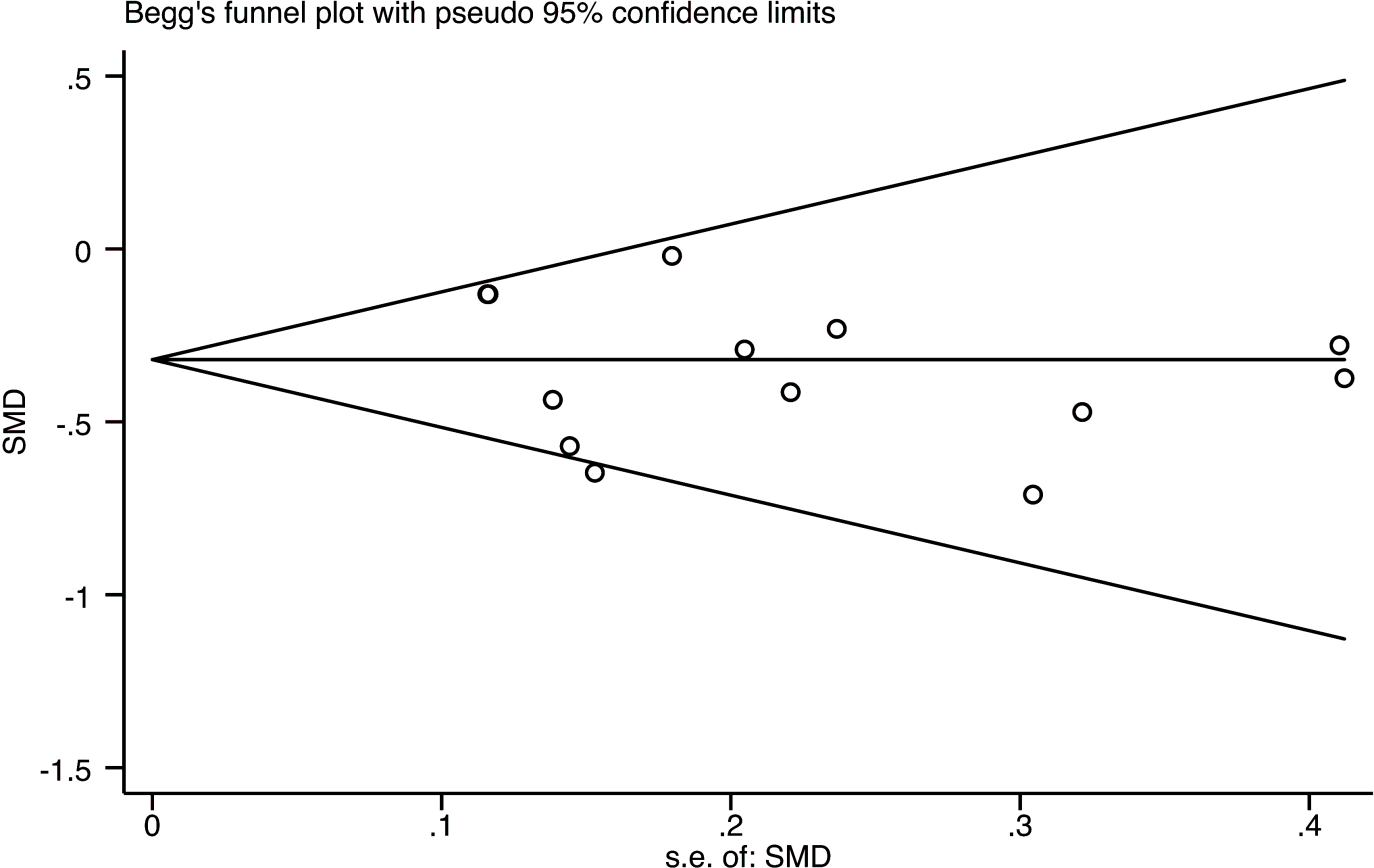
Funnel plot of the effect of mindfulness-based stress reduction (MBSR) on fatigue in breast cancer patients.

Sensitivity analysis and publication bias: Sensitivity analysis of higher heterogeneity was carried out by Stata software, and the analysis results were found to be stable, as shown in **Figure 5**. The publication bias analysis was performed on the main outcome indicator CRF, and a funnel plot was constructed. The results showed that the effect points included in the study were basically funnel-shaped and distributed symmetrically, as shown in **Figure 6**. Combined with the Egger test, the funnel plot was generated, and the results of the Egger test were p = 0.402 > 0.05, which was not statistically significant, indicating that there was no obvious publication bias in this study.

### 3.4 Adverse reactions

Only one study reported adverse effects of this disorder, and no serious adverse effects were reported in this study (**Table 1**).

## 4 Discussion

This systematic study critically analyzed and explained the results of RCTs, and evaluated the effect of MBSR intervention on fatigue during early and mid-term follow-up in breast cancer survivors. Compared with conventional care, MBSR significantly reduced fatigue symptoms (early SMD = -0.32, 95% CI: -0.42 to -0.22; midterm SMD = -0.19, 95% CI: -0.31 to -0.07). The meta-analysis of Xie et al., 2020 included 7 studies on the impact of breast cancer MBSR on CRF, 6 of which were also included in this study, and 1 was not included because the experimental design was not an RCT (38). The early results of MBSR on CRF for breast cancer in this study were similar to those of Zhang and Xie et al., who showed that MBSR has a moderate effect on CRF (20, 38). The reason may be that the patients included in this study were all women who are easily affected by other factors; thus, their fatigue scores fluctuate greatly. However, the mid-term results of MBSR on CRF are inconsistent with those of Haller et al., who reported that the mid-term effect of MBSR on fatigue in breast cancer patients was not statistically significant (21). This may be attributed to the fact that the mid-term follow-up time of the present study ranged from 3 months to 6 months, whereas the follow-up time of two breast cancer-related studies included by Haller et al. was 6 months, which may have led to a reduced effect of MBSR on the fatigue symptoms of breast cancer patients (21). Therefore, the mid-term effect on fatigue in breast cancer patients also needs to be further verified.

The results of the subgroup analysis showed that the fatigue scores of the intervention group were lower than those of the control group after MBSR for 8 weeks or 6 weeks, but MBSR for 8 weeks had a great influence on fatigue symptoms. Future studies should lengthen the intervention period to explore the influence of different intervention durations on fatigue symptoms. The subgroup analysis of intervention methods showed that expert guidance alone was better than expert guidance combined with a compact disc. The possible reason is that the specific method of using compact discs for practice has not been described in detail in the study of expert guidance combined with compact discs, and the specific situation of MBSR at home and patient compliance are unknown. Subgroup analysis of different regions revealed that MBSR improved CRF symptoms in breast cancer patients of different races and from different regions, but MBSR had the greatest impact on CRF symptoms in the Asian population. Our result was inconsistent with the research results of Lam et al., which may be attributed to the different treatment stages of the subjects (45). The subjects in Europe and North America were those who had finished treatment for some time, while most people in Asia were receiving chemotherapy, radiotherapy, or other treatment. Regarding adverse events in MBSR, only one study reported adverse events (28). This might be due to MBSR being a nondrug treatment, leading researchers to neglect its adverse events. Thus, future studies should also report the adverse effects of MBSR and their severity. Breast cancer survivors receiving endocrine therapy exhibit a range of adverse reactions, with CRF being a common symptom among them. A review suggested that MBSR is effective in treating CRF induced by endocrine therapy in breast cancer survivors (46). Therefore, MBSR should be considered an important treatment option for breast cancer survivors undergoing endocrine therapy, and efforts should be made to enhance its accessibility. Given the greater number of physical and psychological problems caused by treatment, MBSR could better reduce fatigue symptoms during this period.

According to the PICOS procedure, we analyzed published systematic reviews and meta-analyses on the impact of mindfulness-based therapy (MBT) on cancer patients (20, 21, 27, 38), in which the research objects included were not limited to breast cancer patients (27, 38), and mindfulness-based intervention was not limited to MBSR but also involved mindfulness-based cognitive therapy (MBCT) and mindfulness-based art therapy (MBAT). In terms of the control group setting, in addition to routine care and blank controls, the aforementioned studies included Qigong intervention and psychosocial support therapy (20.27). In addition to CRF, the outcome indicators included quality of life and other symptoms (20, 21). The studies included not only RCTs but also cluster randomized controlled trials and randomized crossover trials (21). All the studies included in this meta-analysis were RCTs. By comparing the influence of MBSR and routine nursing on the CRF of breast cancer patients, our study confirmed that MBSR can alleviate the CRF of breast cancer patients and revealed that different regions, intervention times, intervention methods and scales affect the intervention effect of MBSR on the CRF of breast cancer patients, which provides a more scientific and accurate evidence-based basis for the clinical symptom management of the CRF of breast cancer patients.

## 5 Limitations

Although we have conducted a comprehensive evaluation of the impact of MBSR intervention on CRF in breast cancer patients, there are still some limitations in the interpretation of the results of this study. First, this study included only Chinese (n = 4) and English (n = 9) studies, and no studies in other languages were retrieved. There may be inevitable selection bias, which will affect the results of this study. Second, among the 13 papers included in the present study, 4 had a sample size of less than 50. Third, there are many factors influencing the CRF level, such as population size, medical/economic conditions, and cognitive/emotional and biological factors. However, the included RCTs did not report the changes in these factors in detail, which may have affected the results. Fourth, there are many kinds of assessment scales for fatigue based on outcome indicators, but the standards are not uniform, which may be the cause of heterogeneity. In the future, it is necessary to develop a special scale to assess the fatigue of breast cancer patients. Fifth, due to the limitations of the original data, there was no subgroup analysis of cancer stage, treatment method, duration, or frequency of each intervention, and the long-term effect of MBSR on CRF was not analyzed. Therefore, it is impossible to propose suggestions for these aspects of MBSR. In the future, further research is needed to extend the follow-up time and carry out multicenter, large-sample randomized controlled trials to provide more specific and detailed guidance for clinical application. Although our systematic review results are positive, the impact is relatively small. Future research should pay more attention to research design, such as randomization, allocation concealment, and blinding, to provide more insights and evidence on MBSR’s CRF for breast cancer.

## 6 Conclusions and implications

Taken together with all the important parameters, MBSR contributes to improving cancer-related fatigue in breast cancer patients and can be recommended as a beneficial adjuvant therapy for cancer-related fatigue in clinical breast cancer patients. In particular, for breast cancer patients currently receiving anticancer treatment, MBSR once a week for two hours for eight weeks, which is supervised by experts, has a significant impact on CRF. Patient compliance plays an important role in the effect of MBSR. Therefore, how to improve patient compliance should be further explored in future clinical CRF management.

## Data availability statement

The original contributions presented in the study are included in the article/**Supplementary Material**. Further inquiries can be directed to the corresponding author.
