# Supplementary material for: Effects of mindfulness-based stress reduction on cancer-related fatigue in patients with breast cancer: a meta-analysis of randomized controlled trials
Source: Front Oncol. 2024 Oct 3;14:1425563. doi: 10.3389/fonc.2024.1425563 (PMC11484075; doi:10.3389/fonc.2024.1425563)
Supplement: Supplementary file 1 [file DataSheet1.docx]

Supplementary Material

# Search strategy

"Breast Neoplasms"[Mesh]

Breast Neoplasm

Neoplasm, Breast

Breast Tumors

Breast Tumor

Tumor, Breast

Tumors, Breast

Neoplasms, Breast

Breast Cancer

Cancer, Breast

Mammary Cancer

Cancer, Mammary

Cancers, Mammary

Mammary Cancers

Malignant Neoplasm of Breast

Breast Malignant Neoplasm

Breast Malignant Neoplasms

Malignant Tumor of Breast

Breast Malignant Tumor

Breast Malignant Tumors

Cancer of Breast

Cancer of the Breast

Mammary Carcinoma, Human

Carcinoma, Human Mammary

Carcinomas, Human Mammary

Human Mammary Carcinomas

Mammary Carcinomas, Human

Human Mammary Carcinoma

Mammary Neoplasms, Human

Human Mammary Neoplasm

Human Mammary Neoplasms

Neoplasm, Human Mammary

Neoplasms, Human Mammary

Mammary Neoplasm, Human

Breast Carcinoma

Breast Carcinomas

Carcinoma, Breast

Carcinomas, Breast

("Breast Neoplasms"[Mesh]) OR (((((((((((((((((((((((((((((((((((((Breast Neoplasm) OR (Neoplasm, Breast)) OR (Breast Tumors)) OR (Breast Tumor)) OR (Tumor, Breast)) OR (Tumors, Breast)) OR (Neoplasms, Breast)) OR (Breast Cancer)) OR (Cancer, Breast)) OR (Mammary Cancer)) OR (Cancer, Mammary)) OR (Cancers, Mammary)) OR (Mammary Cancers)) OR (Malignant Neoplasm of Breast)) OR (Breast Malignant Neoplasm)) OR (Breast Malignant Neoplasms)) OR (Malignant Tumor of Breast)) OR (Breast Malignant Tumor)) OR (Breast Malignant Tumors)) OR (Cancer of Breast)) OR (Cancer of the Breast)) OR (Mammary Carcinoma, Human)) OR (Carcinoma, Human Mammary)) OR (Carcinoma, Human Mammary)) OR (Human Mammary Carcinomas)) OR (Mammary Carcinomas, Human)) OR (Human Mammary Carcinoma)) OR (Mammary Neoplasms, Human)) OR (Human Mammary Neoplasm)) OR (Human Mammary Neoplasms)) OR (Neoplasm, Human Mammary)) OR (Neoplasms, Human Mammary)) OR (Mammary Neoplasm, Human)) OR (Breast Carcinoma)) OR (Breast Carcinomas)) OR (Carcinoma, Breast)) OR (Carcinomas, Breast))

"Mindfulness"[Mesh]

Mindfulness

mindfulness based stress reduction

mbsr

meditation

relaxation therapy

mind-body therapies

body-mind

mind-body near

Mindfulness-Based Stress Reduction

("Mindfulness"[Mesh]) OR (((((((((Mindfulness) OR (mindfulness based stress reduction)) OR (mbsr)) OR (meditation)) OR (relaxation therapy)) OR (mind-body therapies)) OR (body-mind)) OR (mind-body near)) OR (Mindfulness-Based Stress Reduction))

"Fatigue"[Mesh]

Fatigue

Lassitude

fatigue cancer related

cancer related fatigue

Asthenia

Neurasthenia

Muscle Weakness

fatigue syndrome

muscle fatigue

("Fatigue"[Mesh]) OR (((((((((Fatigue) OR (Lassitude)) OR (fatigue cancer related)) OR (cancer related fatigue)) OR (Asthenia)) OR (Neurasthenia)) OR (Muscle Weakness)) OR (fatigue syndrome)) OR (muscle fatigue))

(((("Breast Neoplasms"[Mesh]) OR (((((((((((((((((((((((((((((((((((((Breast Neoplasm) OR (Neoplasm, Breast)) OR (Breast Tumors)) OR (Breast Tumor)) OR (Tumor, Breast)) OR (Tumors, Breast)) OR (Neoplasms, Breast)) OR (Breast Cancer)) OR (Cancer, Breast)) OR (Mammary Cancer)) OR (Cancer, Mammary)) OR (Cancers, Mammary)) OR (Mammary Cancers)) OR (Malignant Neoplasm of Breast)) OR (Breast Malignant Neoplasm)) OR (Breast Malignant Neoplasms)) OR (Malignant Tumor of Breast)) OR (Breast Malignant Tumor)) OR (Breast Malignant Tumors)) OR (Cancer of Breast)) OR (Cancer of the Breast)) OR (Mammary Carcinoma, Human)) OR (Carcinoma, Human Mammary)) OR (Carcinoma, Human Mammary)) OR (Human Mammary Carcinomas)) OR (Mammary Carcinomas, Human)) OR (Human Mammary Carcinoma)) OR (Mammary Neoplasms, Human)) OR (Human Mammary Neoplasm)) OR (Human Mammary Neoplasms)) OR (Neoplasm, Human Mammary)) OR (Neoplasms, Human Mammary)) OR (Mammary Neoplasm, Human)) OR (Breast Carcinoma)) OR (Breast Carcinomas)) OR (Carcinoma, Breast)) OR (Carcinomas, Breast))) AND (("Mindfulness"[Mesh]) OR (((((((((Mindfulness) OR (mindfulness based stress reduction)) OR (mbsr)) OR (meditation)) OR (relaxation therapy)) OR (mind-body therapies)) OR (body-mind)) OR (mind-body near)) OR (Mindfulness-Based Stress Reduction)))) AND (("Fatigue"[Mesh]) OR (((((((((Fatigue) OR (Lassitude)) OR (fatigue cancer related)) OR (cancer related fatigue)) OR (Asthenia)) OR (Neurasthenia)) OR (Muscle Weakness)) OR (fatigue syndrome)) OR (muscle fatigue)))) AND (randomized controlled trial[Publication Type] OR randomized[Title/Abstract] OR placebo[Title/Abstract])
